# Pathways to “5-a-day”: modeling the health impacts and environmental footprints of meeting the target for fruit and vegetable intake in the United Kingdom

**DOI:** 10.1093/ajcn/nqab076

**Published:** 2021-04-19

**Authors:** Patricia Eustachio Colombo, James Milner, Pauline F D Scheelbeek, Anna Taylor, Alexandr Parlesak, Thomas Kastner, Owen Nicholas, Liselotte S Elinder, Alan D Dangour, Rosemary Green

**Affiliations:** Department of Global Public Health, Karolinska Institutet, Stockholm, Sweden; Centre on Climate Change and Planetary Health, London School of Hygiene and Tropical Medicine, London, United Kingdom; Centre on Climate Change and Planetary Health, London School of Hygiene and Tropical Medicine, London, United Kingdom; The Food Foundation, London, United Kingdom; Global Nutrition and Health, University College Copenhagen, Copenhagen, Denmark; Senckenberg Biodiversity and Climate Research Centre, Frankfurt, Germany; Department of Statistical Science, University College London, London, United Kingdom; Department of Global Public Health, Karolinska Institutet, Stockholm, Sweden; Centre on Climate Change and Planetary Health, London School of Hygiene and Tropical Medicine, London, United Kingdom; Centre on Climate Change and Planetary Health, London School of Hygiene and Tropical Medicine, London, United Kingdom

**Keywords:** planetary health, dietary patterns, health impact assessment, nutrition, health promotion, diet cost

## Abstract

**Background:**

Fruit and vegetable consumption in the United Kingdom is currently well below recommended levels, with a significant associated public health burden. The United Kingdom has committed to reducing its carbon emissions to net zero by 2050, and this transition will require shifts towards plant-based diets.

**Objective:**

The aim was to quantify the health effects, environmental footprints, and cost associated with 4 different pathways to meeting the United Kingdom's “5-a-day” recommendation for fruit and vegetable consumption.

**Methods:**

Dietary data based on 18,006 food diaries from 4528 individuals participating in the UK National Diet and Nutrition Survey (2012/13–2016/17) constituted the baseline diet. Linear programming was used to model the hypothetical adoption of the 5-a-day (400 g) recommendation, which was assessed according to 4 pathways differing in their prioritization of fruits versus vegetables and UK-produced versus imported varieties. Increases in fruit and vegetable consumption were substituted for consumption of sweet snacks and meat, respectively. Changes in life expectancy were assessed using the IOMLIFET life table model. Greenhouse gas emissions (GHGEs), blue water footprint (WF), and total diet cost were quantified for each 5-a-day diet.

**Results:**

Achieving the 5-a-day target in the United Kingdom could increase average life expectancy at birth by 7–8 mo and reduce diet-related GHGEs by 6.1 to 12.2 Mt carbon dioxide equivalents/y; blue WFs would change by −0.14 to +0.07 km^3^/y. Greater reductions in GHGEs were achieved by prioritizing increased vegetable consumption over fruit, whereas the greatest reduction in WF was obtained by prioritizing vegetable varieties produced in the United Kingdom. All consumption pathways increased diet cost (£0.34–£0.46/d)

**Conclusions:**

Benefits to both population and environmental health could be expected from consumption pathways that meet the United Kingdom's 5-a-day target for fruit and vegetables. Our analysis identifies cross-sectoral trade-offs and opportunities for national policy to promote fruit and vegetable consumption in the United Kingdom.

## Introduction

The United Kingdom has committed in law to cut greenhouse gas emissions (GHGEs) and become a net-zero carbon economy by 2050 (1). Accelerating shifts towards healthier and more sustainable diets in order to reduce the environmental impact of the UK food system has been identified as 1 of 6 strategies critical to reaching the net-zero target (2). The necessary dietary shifts are likely to involve replacing a proportion of the current consumption of animal-source foods with plant-based foods (3–5). Diets high in fruit and vegetables (including legumes) typically have a lower environmental footprint (6, 7), although this is not always the case (8, 9), and the impacts are influenced by local agricultural, food system, and environmental contexts (10).

The United Kingdom's Eatwell Guide and national “5-a-day” campaign already recommends the consumption of 5 portions of a variety of fresh, canned, or frozen fruit and vegetables (including legumes) every day (11, 12). A recent study reported that this campaign (initiated in 2003) had increased fruit and vegetable consumption by approximately half a portion daily 10 y after the policy launch (13). Complementary findings show that the supply of fruits and vegetables to the UK population has increased, while the supply of animal-source foods and sugar has declined over the last 40 y (14). Despite these changes, mean fruit and vegetable intakes in the United Kingdom remain well below the 5-a-day recommendation across all age and sex groups (15). There is convincing evidence that low consumption of fruit and vegetables is a strong risk factor for noncommunicable diseases (NCDs) (16) and this is particularly concerning since diet-related NCDs are one of the leading causes of death in the UK population (17). Around 7% of disability-adjusted life-years lost in the United Kingdom were attributed to diets low in fruits or vegetables in 2010, a larger proportion of the total burden of disease than physical inactivity or alcohol use (18).

Similar to other northern European countries, the United Kingdom is currently heavily reliant on international trade for supplying fruit and vegetables (19) and many supplier countries are becoming increasingly vulnerable to the adverse effects of environmental change (19, 20). Continued reliance on imported produce might not only exacerbate conditions in supplier countries (by, e.g., contributing to water stress) but also make the United Kingdom more vulnerable to market and price volatilities (19). Consumers’ abilities to purchase fruit and vegetables—foods with high price elasticities (21)—may be significantly jeopardized with increased reliance on fruit and vegetable imports, especially under a potential no-deal Brexit (22). To support the UK population to further increase fruit and vegetable intakes will, among other things, require national policies that help ensure affordable and stable fruit and vegetable supplies that are resilient to future political and/or environmental change.

Ideally, food policy changes in times of climate disruption need to be informed by evidence concerning both proximal (e.g., dietary) and distal (e.g., health/environmental) outcomes of food systems (23). In this diet-modeling study, we thus aimed to develop 4 hypothetical consumption pathways to reach the United Kingdom's 5-a-day target for fruit and vegetable intake on a national scale. We sought to quantify the potential effects on health (changes to years of life gained, life expectancy at birth), environmental footprints [GHGEs and blue water footprints (WFs)], and dietary cost that could be expected from these consumption pathways. We included consideration of different fruit and vegetable varieties, their countries of origin, as well as various options for dietary substitutions.

## Methods

### Data

#### Population sample and dietary data

Dietary data were derived from the self-reported intake over 4 d of an area-stratified random sample of the UK population participating in the National Diet and Nutrition Survey (NDNS) waves 5–9 (2012/13–2016/17) (24). The NDNS is a rolling program of cross-sectional surveys based on a 4-d food diary. These data were chosen as they presently constitute the only nationally representative dietary intake data for the UK population. The NDNS data provide quantities (in grams) of items eaten or drunk over 4 consecutive days, per main food group (e.g., “fruit”), subfood group (e.g., “bananas”), and per individual (discrete) food item (e.g., “bananas raw”) (24). Information from Public Health England's Nutrient Databank (NDB) (25) provides the NDNS data with the energy and nutrient composition of the edible share of each dietary entry made by the survey participants. For this analysis, dietary data based on 18,006 food diaries from 4528 individuals aged 12–95 y and reported over 3 or 4 d were used (**Supplemental Figure 1** and **Supplemental Table 1**). These were aggregated to quantify the total national consumption (grams) and energy content (kilocalories) of 265 new compositionally distinct food groups, of which 48 contained fruits, 64 contained vegetables, and 153 contained all other types of foods (full list of compositionally distinct food groups available in **Supplemental Data 1**). The fruit and vegetable food groups were not aggregated as much as the other foods in order to provide a more detailed overview of the reported intakes of different varieties of fruit and vegetables and to facilitate comparisons between them in terms of sustainability (**Supplemental Tables 3 and 4**). The total consumption and energy content of each of the 265 food groups were averaged (divided by the 18,006 reporting days) to provide an average national daily intake. To provide a range for plausible food intakes, 95% CIs for the average daily intakes of all 265 food groups were calculated.

For the purpose of this study, fruit and vegetables counting towards the 5-a-day target excluded fruit juices and potatoes. The Eatwell Guide recommends an intake of ≥5 portions of fruit and vegetables including legumes and canned products (26). Fresh, canned, frozen, and dried fruits and vegetables were thus all included in varieties counting towards the 5-a-day target. Legumes, which were included in the vegetable category, were limited to 1 portion (i.e., 80 g) of the 5-a-day as recommended in the Eatwell Guide (26), and fruit juice was excluded from the fruit portions due to concerns over sugar content, which is found to increase the risk for type 2 diabetes (27). Potatoes were excluded since they are considered a starchy carbohydrate food (12). Total fruit and vegetable consumption considered all fruit and vegetable types reported as discrete items in the NDNS survey. Hence, small amounts of fruit and vegetables contained within highly processed foods (e.g., fruit yogurts, cereal bars, and ready meals) were excluded from the target.

#### GHGEs, blue WF, and cost of foods

The environmental impacts assessed in this study were the diet-related GHGEs and blue WF—water from ground and surface reserves only as opposed to water absorbed through precipitation—used in the production of each food group. The GHGEs of the foods were expressed as carbon dioxide equivalents (CO_2_eq), including emissions of carbon dioxide (CO_2_), methane (CH_4_), and nitrous oxide (N_2_O). These data were based on outputs from partial or complete Life Cycle Assessments (28, 29) and compiled from published literature (30–35). Besides GHGEs, we chose to only consider the blue WF as this is a natural resource that can be depleted, and thus commonly used to assess the anthropogenic WF from food production (10). The analyses estimated UK-specific environmental footprints as well as country-specific values corresponding to the footprints of imported goods according to their most common countries of origin (35). An amended trade database from the FAO including information on the primary origins of foods consumed in the United Kingdom, as well as data from the Department for Environment, Food and Rural Affairs (36), providing information on the proportion of fruit and vegetable types imported versus locally produced, were used for the compilation of the environmental impact values (Supplemental Data 1). Global average data were used when country-specific estimates of environmental footprints were not available. The environmental footprint and trade data were combined to calculate environmental impact values for each food group and each individual fruit or vegetable that were weighted according to current supply patterns (Supplemental Data 1 and **Supplemental Table 5**).

To estimate changes in the cost of diet, food prices for individual items were included. These were collected online (during the period February–April 2018) at mysupermarket.co.uk, representing a number of UK supermarkets including Tesco, Asda, Ocado, Waitrose, Sainsbury's, Morrisons, Poundland, Coop, Iceland, Aldi, and Lidl. In total, 14,686 prices for 7583 food products were collected and were adjusted for the weight of individual food items. The average of all collected prices per food (UK pounds per kilogram) was used as the price during optimization (Supplemental Data 1).

### Pathways to 5-a-day

**FIGURE 1:**
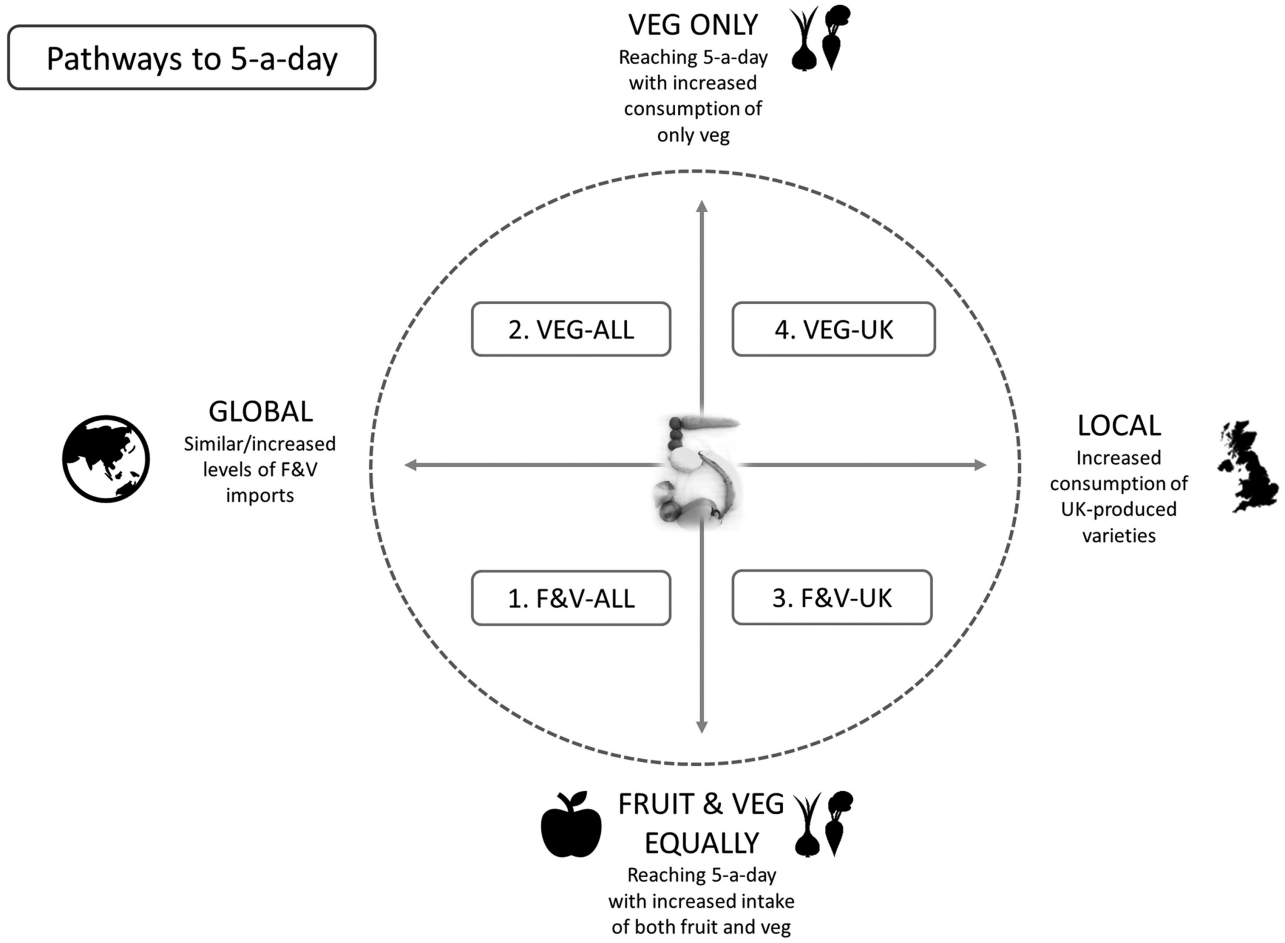
Four pathways to 5-a-day. F&V, fruit and vegetables; veg/VEG, vegetables.

We developed 4 possible consumption pathways to achieve the 5-a-day recommendation for fruit and vegetable intake in the United Kingdom (**Supplemental Table 6**). In all 4 pathways, total fruit and vegetable consumption per person was increased from current levels to 5 portions (400 g) per day. To attain this level of fruit and vegetable consumption, we defined pathways focusing on 2 current areas of health and environmental policy debate: *1*) whether consumption of vegetables should be prioritized over fruit due to their lower sugar content and their generally lower environmental footprints (10, 37, 38) and *2*) whether consumption of fruit and vegetables produced in the United Kingdom should be prioritized over imported fruit and vegetable varieties to support national food security and resilience to climate change (20, 31, 32) (**Figure 1**).

The 4 hypothetical consumption pathways are as follows:

1. F&V-ALL: the additional consumption required to achieve 5-a-day comes from all fruit and vegetable varieties (proportionate to their current consumption in the United Kingdom) and is sourced from the same countries as current patterns.
2. VEG-ALL: the additional consumption required to achieve 5-a-day comes only from all vegetable varieties (proportionate to their current consumption in the United Kingdom) and is sourced from the same countries as current patterns.
3. F&V-UK: the additional consumption required to achieve 5-a-day comes only from fruit and vegetable varieties that could plausibly be grown to a greater extent in the United Kingdom (i.e., “UK-capable crops”) (**Supplemental Table 7**), proportionate to their current consumption in the United Kingdom.
4. VEG-UK: the additional consumption required to achieve 5-a-day comes only from vegetable varieties that could plausibly be grown to a greater extent in the United Kingdom (i.e., “UK-capable crops”) (Supplemental Table 7), proportionate to their current consumption in the United Kingdom.

Evidence from fruit and vegetable intervention and modeling studies concerning what happens to consumption of other foods is sparse but recent analyses of food consumption trends in the United Kingdom show that intakes of animal-source foods and sugar have declined while intakes of fruits, and vegetables in particular, have increased over the last 40 y (20). Furthermore, several studies indicate that fruit consumption can replace consumption of sweet snacks (39, 40), and modeling studies also indicate that vegetables/legumes are a plausible substitute for meat (41, 42). Assumptions about plausible food substitutions were made on the basis of these findings. Therefore, in all 4 hypothetical pathways modeled, additional fruit consumption replaced consumption of sweet snacks and additional vegetable consumption replaced consumption of meat (red, processed, and poultry) on a per-kilocalorie basis (Supplemental Table 6).

Linear programming was used to model the dietary modifications proposed by each hypothetical pathway and to constrain the dietary energy content so that average energy content was equal to that in the observed diets (1744 kcal/d) (**Supplemental Table 2 and Supplemental Figure 2**). Average dietary energy intake was held constant so that any differences observed in health effects or environmental footprints would be due to the change in dietary pattern rather than a change in total dietary energy intake. The total relative deviation from the current UK diet was minimized across all food groups that were not directly modified (i.e., all foods besides fruits, vegetables, meat, and sweet snacks). This was done to keep dietary patterns in the optimized pathways as similar as possible to baseline food patterns. Linear programming was performed using the CBC (COIN-OR Branch and Cut) Solver algorithm, which is part of the Microsoft Excel 2016 software add-in OpenSolver, version 2.9.0 (43). More information on the optimization procedure can be found in the Supplemental data.

### Outcomes

#### Modeling the health impacts

Health impacts were the main outcome of this study and calculated on the basis of a previously applied methodology (44), using the life table model IOMLIFET (45) implemented in R (46). Briefly, the IOMLIFET model estimates survival patterns in the population over time based on age-specific mortality rates. Based on the information of a hypothetical change in diet (risk exposure) and a known exposure–response function, changes in survival rates can be quantified as, for example, years of life lost (YLL) or changes to life expectancy. YLL can be explained as the years of life lost for an individual (or a population) as a result of premature avertable mortality, considering the age at which deaths occurred.

In this study, we quantified changes in YLL and average life expectancy in the United Kingdom as a result of modifications in the consumption of fruits, vegetables, legumes, red meat, and processed meat resulting from the 4 consumption pathways. Since these dietary modifications were expected to reduce mortality rates, YLL were translated to years of life gained.

Dose–response relations (i.e., RRs) between dietary intake and mortality from chronic diseases were obtained from the latest Global Burden of Disease (GBD) study (37). The RR for a dietary risk–disease pair shows how much the risk of mortality (or morbidity) would change when the dietary risk changes. For example, the risk of ischemic heart disease is reduced by 14% for each 100-g increase in fruit intake (**Table 1**). We studied the following disease endpoints: ischemic heart disease, ischemic stroke, type 2 diabetes, and site-specific cancers (Table 1 and **Supplemental Table 8**). These endpoints were selected as they currently contribute most to diet-related life-years lost in the United Kingdom (47). For the health impact modeling, changes in legumes (included in the vegetables category in the optimized dietary pathways) were calculated separately since the exposure–response relations for legumes differ from those of other vegetables (Table 1). Also, although the consumption pathways enforced reductions in all meat products as a result of increased vegetable intakes, the health impact calculations only encompassed dietary changes in processed meat and red meat—that is, not including changes in unprocessed poultry meat, due to a lack of data on RRs from changes in poultry consumption.

**TABLE 1:** Dietary exposure–response pathways (including upper and lower 95% CIs) used in the health impact modeling

Dietary exposure and health outcome	Unit	RR^1^	95% CI
Fruit			
Ischemic heart disease	100-g increase	0.86	(0.79, 0.95)
Ischemic stroke	100-g increase	0.65	(0.55, 0.79)
Tracheal, bronchus, and lung cancer	100-g increase	0.93	(0.89, 0.97)
Esophageal cancer	100-g increase	0.87	(0.78, 0.97)
Type 2 diabetes	100-g increase	0.91	(0.84, 0.98)
Vegetables			
Ischemic heart disease	100-g increase	0.86	(0.78, 0.94)
Ischemic stroke	100-g increase	0.87	(0.79, 0.97)
Legumes			
Ischemic heart disease	50-g increase	0.76	(0.65, 0.89)
Red meat			
Colorectal cancer	100-g decrease	0.86	(0.76, 0.97)
Type 2 diabetes	100-g decrease	0.80	(0.68, 0.97)
Processed meat			
Ischemic heart disease	50-g decrease	0.56	(0.39, 0.97)
Colorectal cancer	50-g decrease	0.85	(0.79, 0.91)
Type 2 diabetes	50-g decrease	0.58	(0.47, 0.76)

1 Based on the latest Global Burden of Disease study (37).

Life tables were separately generated for males and females due to their different underlying mortality rates. Age-specific and sex-specific population-size estimates from the Office for National Statistics (48), as well as data on all-cause mortality and disease-specific mortality from the GBD results-tool (49), were combined to create input data for the United Kingdom. Diets were assumed to be adopted instantly while underlying mortality rates remained constant for the duration of follow-up. The exposure–response functions were assumed to be log-linear and, in cases where several dietary exposures affected the same disease, the risks were multiplied together as done previously (44). Changes in life expectancy at birth were calculated as the difference between baseline life expectancy (the expected life-years divided by the starting population) and the impacted (modeled) life expectancy (the impacted expected life-years by the impacted starting population).

Previous research assessing effects of dietary interventions on various causes of mortality has established approximate time lags between exposure and onset of disease (20). Hence, time lags for ischemic heart disease, stroke, and type 2 diabetes were assumed to reach a maximum impact after 10 y and for cancers after 30 y, with no change in cancer risk during the first 10 y. Time-varying functions based on cumulative distribution functions of normally distributed variables (S-shaped curves) were implemented to account for time lags between dietary changes and changes in health outcomes. Details with regard to the implementation of the time lags have previously been described (44).

To test the sensitivity of the health impacts from each pathway, we generated upper and lower health impact estimates using high and low estimates for the RRs based on the 95% CI from the GBD study (37) (Table 1).

#### Total GHGEs, blue WF, and cost of observed and optimized dietary pathways

The overall GHGEs (in kilograms CO_2_eq) and blue WF (in total liters of freshwater from ground and surface sources) of the current mean UK diet and the four 5-a-day pathways were the secondary outcomes and calculated as the sum of the corresponding reported food weights multiplied by their specific CO_2_eq and WF values as recorded in the literature (Supplemental Data 1). The total weight of each food item was multiplied by the specific cost of the product as consumed to obtain the cost of the observed and optimized diets, respectively. Diet cost was also a secondary outcome.

## Results

The observed UK diet for the years 2011–2017 among individuals aged ≥12 y contained an average of 88 g (just over 1 portion) of fruit per day and an average of 140 g (just under 2 portions) of vegetables per day. The main types of fruits were bananas, apples, pears, and citrus fruits and the main types of vegetables were tomatoes, baked beans, onions, carrots, and peas (**Table 2**).

**TABLE 2:** Main types of fruit and vegetables (providing 90% of their respective category) in the observed UK diet, including daily amounts (grams) and proportion (%) of total baseline amounts of fruit or vegetables

Fruit	Amount, g	Proportion of total (88 g), %	Vegetable	Amount, g	Proportion of total (140 g), %
Bananas	23.84	27	Tomatoes	24.93	18
Apples	18.27	21	Baked beans^1^	15.47	11
Pears	5.64	6	Onions	13.88	10
Oranges	5.32	6	Carrots	13.57	10
Tangerines	5.32	6	Peas	8.18	6
Grapes	5.12	6	Broccoli	6.57	5
Strawberries	4.72	5	Peppers	5.85	4
Canned fruit	1.91	2	Cucumber	4.83	3
Melons	1.83	2	Mushrooms	4.56	3
Pineapple	1.61	2	Lettuce	4.14	3
Blueberries	1.44	2	Cabbage	3.72	3
Mangoes	1.43	2	Cauliflower	2.93	2
Nectarines	1.29	1	Green beans	2.74	2
Plums	1.20	1	Other beans	2.47	2
			Sweet corn	2.46	2
			Sweet potato	1.63	1
			Lentils	1.55	1
			Spinach	1.51	1
			Parsnips	1.50	1
			Mixed-leaf salad	1.44	1
			Leeks	1.42	1

1 In the United Kingdom consumed as a dish containing white beans, tomatoes, and water as main ingredients.

Mean dietary energy intake from the whole diet was 1744 kcal per person per day (1750 kcal and 1726 kcal per person per day for adults and children, respectively; Supplemental Table 2). The mean cost of the diet was £6.78 per person per day; mean GHGEs were 6.2 kg CO_2_eq per person per day (**Table 3**), of which 1.6% and 3.9% derived from fruit and vegetables, respectively (**Supplemental Figure 3**); and mean blue WF was 611 L per person per day (Table 3), of which 0.9% and 1.0% derived from fruit and vegetables, respectively (Supplemental Figure 3). Aggregating these average daily amounts of CO_2_eq and WFs over 1 y and for a UK population of 66.65 million would amount to ∼148 Mt CO_2_eq and 14.7 km^3^ of blue water for total annual food consumption.

**TABLE 3:** Changes in environmental footprints, cost, and health impacts for each of the pathways to 5-a-day compared with current UK consumption^1^

Diet impacts^2^ and units	Current diet	F&V-ALL	VEG-ALL	F&V-UK	VEG-UK
Environmental footprints^3^					
CO_2eq_/person per day					
kg	6.2	5.9	5.7	5.9	5.7
Change in kg (%)	NA	−0.3 (−4.1)	−0.5 (−8.2)	−0.3 (−4.8)	−0.4 (−7.0)
WF/person per day					
L	611.4	614.2	610.5	605.6	605.4
Change in L (%)	NA	2.8 (0.5)	−0.9 (−0.2)	−5.8 (−0.9)	−6.0 (−1.0)
Diet cost^3^					
Diet cost/person per day					
GBP	6.78	7.14	7.12	7.24	7.21
Change in GBP (%)	NA	0.36 (4.4)	0.34 (4.4)	0.46 (5.9)	0.43 (5.9)
Health impacts^4^					
Life expectancy					
Years (mo)	81.1 (973)^5^	81.8 (981)	81.8 (981)	81.7 (980)	81.7 (980)
Change in months	NA	8.0	8.2	7.4	7.3
% attributed to fruit and/or vegetables^6^	NA	83	75	83	77

^1^CO_2eq_, carbon dioxide equivalents; F&V, fruit and vegetables; GBP, Great Britain Pound (1 GBP = ∼1.3 US dollar); NA, not applicable; VEG, vegetables; WF, water footprint.

2 CIs for the diet impacts are found in Supplemental Tables 9 and 12.

3 Quantified for the baseline diet extracted as an output from the optimization models.

4 Assessed using life table models.

5 Average life expectancy at birth of the baseline population (81.07 y).

6 Share (%) of the change in average life expectancy attributed to increased fruit and/or vegetable consumption only.

**FIGURE 2:**
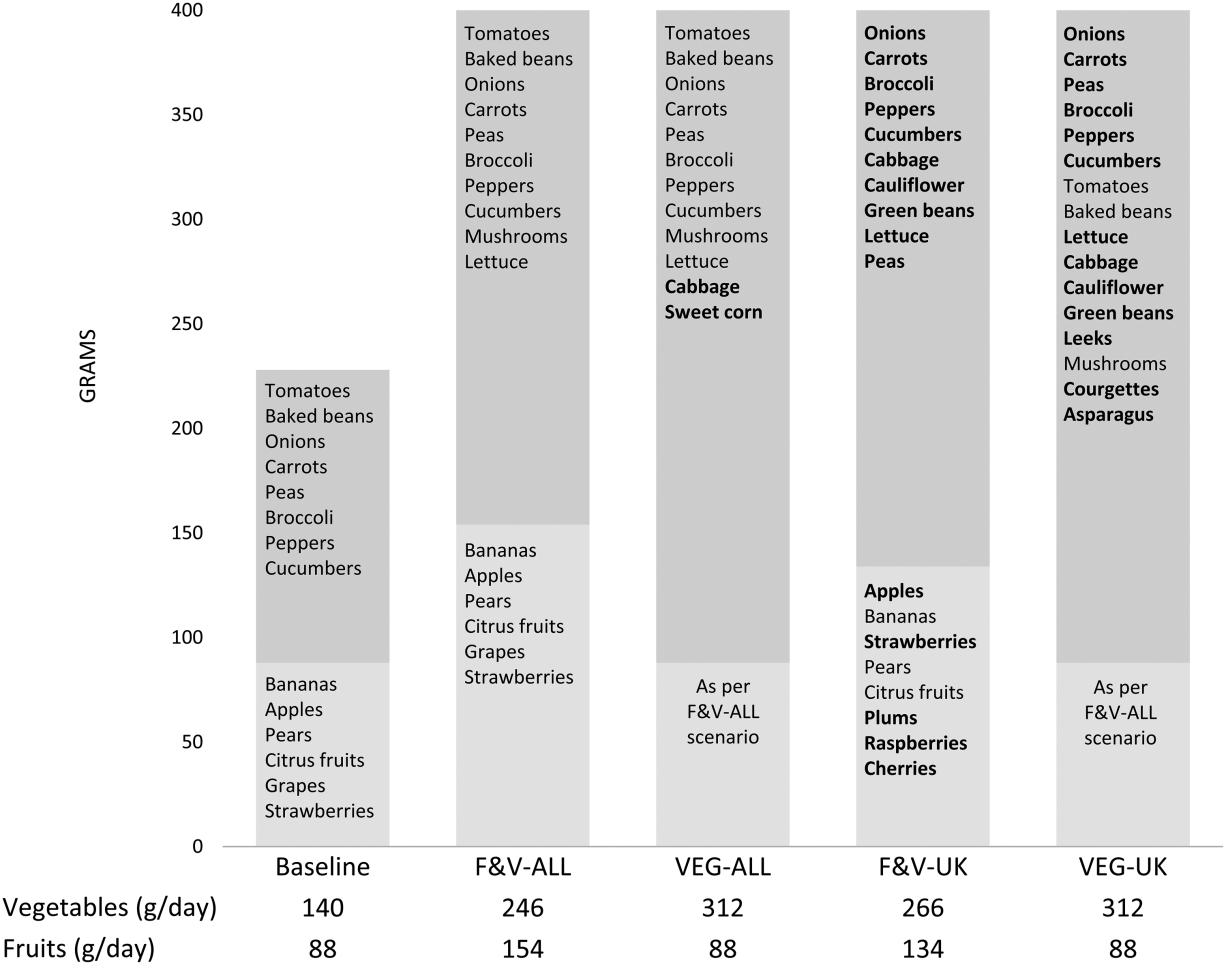
The absolute amounts (g/d) of fruit and vegetables in the baseline diet and 4 optimized pathways, including information on the main contributing fruits and vegetable crops (in descending order) for each model. Bolded fruit and vegetable varieties are those with a substantial increase from current UK consumption. The 95% CI for absolute amounts (g/d) of fruit and vegetables in the baseline diet and 4 optimized pathways are shown in Supplemental Table 9. F&V, fruit and vegetables; VEG, vegetables.

In the 4 modeled diets, vegetable consumption increased from a baseline average of 140 g/d to between 246 g/d (a 76% increase) and 312 g/d (a 123% increase), with the greatest consumption being in the VEG-ALL and VEG-UK pathways (**Figure 2**). Fruit consumption increased from a baseline average of 88 g/d to 154 g/d (a 75% increase) in the F&V-ALL pathway and 134 g/d (a 52% increase) in the F&V-UK pathway, but remained the same in the 2 vegetable-only pathways. Of the 5-a-day portions, 2 came from fruit and 3 from vegetables in the “ALL” diets, whereas 1 portion came from fruit and 4 from vegetables in the “VEG” diets. The most popular varieties were baked beans and bananas in the current diet and the F&V-ALL and VEG-ALL pathways. The UK-focused diets increased consumption over a wider range of fruit and vegetables than the “ALL” diets, with baked beans and bananas replaced by onions and apples as the most popular varieties.

The effects on health were positive in all 4 pathways, resulting in approximately an 8-mo increase in life expectancy for the F&V-ALL and VEG-ALL diets and a 7-mo increase for the F&V-UK and VEG-UK diets (Table 3). Approximately 80% of the health gains were attributable to health improvements from increased consumption of fruit and vegetables and the remainder was attributable to decreased consumption of red and processed meat. The sensitivity analyses of the health impacts from each pathway (**Supplemental Tables 10–12**) provide an indication of the ranges around our central model estimates.

When compared with current average diets in the United Kingdom, all 4 of the pathways to 5-a-day had reduced total GHGEs from the total diet (Table 3). The biggest reductions were seen in the VEG-ALL pathway (an 8.2% reduction), followed by the VEG-UK pathway (a 7% reduction). These reductions would help to reduce diet-related GHGEs by 6.1–12.2 Mt CO_2_eq/y (depending on the pathway), translating to ∼0.8 to 1.6% of annual GHGEs (50). The greatest reductions in blue WF were observed in the VEG-UK pathway (a 1.0% reduction) and the F&V-UK pathway (a 0.9% reduction). The F&V-ALL pathway increased blue WF by 0.5% (+0.07 km^3^/y). Following the remaining 3 diets would help reduce the WF by 0.02–0.14 km^3^/y in the United Kingdom (depending on pathway), translating to ∼0.3–2.0% of annual blue WF (51) associated with UK consumption (both from imported and domestic production).

GHGEs from fruit and vegetables increased in all 4 modeled diets compared with the baseline diet due entirely to increased consumption. In contrast, blue WFs only appreciably increased in the “ALL” diets that contained more imported varieties due to their higher water use (Supplemental Figure 3).

All 4 of the modeled diets cost more than the current average diet due to a higher average cost of fruit and vegetables per kilocalorie compared with the foods they were replacing, particularly sweet snacks. The F&V-UK and VEG-UK diets were the most expensive, at £7.24 and £7.21 per day, respectively—an increase in the diet cost of nearly 6%.

## Discussion

Our study has revealed notable benefits to population health and the environment associated with 4 different hypothetical consumption pathways to meet the United Kingdom's 5-a-day recommendation for fruit and vegetables. These dietary changes could contribute to 10–31% of the goal to reduce domestic land-based emissions by 37 Mt within 30 y (52). Life expectancy would increase by 8 mo, which corresponds to 13% of the NHS (National Health Service) target to extend average life expectancy in the United Kingdom by 5 y by 2035 (53). Greater reductions in GHGEs were achieved by prioritizing increased vegetables over fruit in the modeled diets, as the “VEG” pathways resulted in greater reductions in meat consumption (**Supplemental Table 9**). This was considered a plausible substitution, since many meat substitutes are vegetable-based and vegetables tend to be consumed as part of a savory meal in contrast to fruit, which is often consumed as a snack. Greater reductions in water use were observed for pathways that prioritized fruit and vegetables produced in the United Kingdom rather than imported varieties, due to the generally lower WF of UK-produced varieties, which rely more on rainfall for their production. Health gains in the 2 “UK” pathways were slightly lower due to UK-produced fruit and vegetables having a lower energy density, on average, therefore requiring a smaller reduction in consumption of meat and sweet snacks to balance out energy intake. The increase in cost was also higher for these 2 pathways due to a higher cost of (“UK-capable”) fruit and vegetable varieties that were increased in these pathways.

To the best of our knowledge, this is the first study to explore the health effects and environmental footprints of different consumption pathways to meeting the United Kingdom's 5-a-day recommendation for fruit and vegetables. Our results tally with previous studies showing lower environmental footprints and improved health outcomes from diets that align with dietary recommendations (35, 44, 54), but crucially add new evidence to support the introduction of specific policies to increase fruit and vegetable consumption. Our results also echo the findings of previous studies that have made the link between reduced imports of fruit and vegetables and reduced carbon footprints from these foods (55). However, our study was able to investigate these co-benefits and trade-offs further by exploring plausible changes to the entire diet and by incorporating data on the predominant countries of origin and production systems used to supply diets in the United Kingdom. Our findings provide valuable insights to the development of environmentally sustainable and healthy national food supply strategies.

This analysis brought together data from a number of sources in order to explore co-benefits and trade-offs of various pathways of increased fruit and vegetable consumption. An important strength is the ability to compare realistic UK diets across dimensions of health, sustainability, and cost, as well as incorporating information on imported versus UK-produced crops. The analysis also has a number of limitations that will limit interpretation, and many of these relate to data availability and quality. The food diary method used in NDNS has been shown to underestimate food consumption (potentially by ∼30%) (56) and may not always represent people's usual dietary patterns. Despite this limitation, the NDNS presently constitutes the only continuous nationally representative dietary data for the UK population. The potential to improve health and reduce environmental footprints may be underestimated in this study due to keeping the total average dietary energy intake constant across pathways. In practice, reductions in dietary energy intake would be desirable in the United Kingdom (57) and the accruing health and environmental benefits would be greater than those we have reported.

Every effort was made to use current, context-specific data on environmental footprints of foods, although it was not always possible to use GHGE or WF estimates that represented the country of origin of a particular food. The environmental footprint data necessarily came from multiple sources and the methods used may not always have been comparable, which will have led to some inaccuracies in estimation. The health impact assessment was necessarily conservative as we focused only on the most robust diet–disease relations and did not include health impacts from changes in consumption of other foods or nutrients—for example, sodium, sugar, or nuts and seeds. Our analysis therefore is likely to have underestimated the health benefits of the dietary substitutions under all 4 pathways. Moreover, the health impact models were based on pooled estimates of health impacts from all fruits or all vegetables combined and therefore cannot distinguish between varieties with slightly different nutrient profiles. It is therefore possible that health benefits for people consuming fruits and vegetables with a higher sugar or sodium content may be less. The extrapolation of our results is also limited by the fact that we did not consider seasonality in the selection of prioritized “UK-capable crops,” most of which cannot be considered year-round produce in the United Kingdom.

We made some necessary assumptions about dietary substitution in our analysis. These assumptions were substantiated by data from previous intervention and modeling studies as well as very recent analyses of food consumption trends in the United Kingdom (58). Despite these observed trends, we cannot definitively ascertain that such substitutions would occur in practice (58). Consumption pathways to 5-a-day that allow for various dietary preferences (e.g., vegetarianism) to be considered should be investigated further.

Our findings of greater benefits from the pathways that prioritized vegetables over fruit substantiate the recommendations of several countries including Sweden, India, and the United States, which have chosen to promote consumption of vegetables over fruit for both health and sustainability reasons (59–61). Recent studies have, however, shown that the UK population increasingly prefers imported tropical fruit over locally grown vegetables (19). These trends may be difficult to reverse without positive interventions on the part of government and the food industry.

Substantial behavior change would be needed to achieve the benefits outlined in this study, particularly given the additional costs of the modeled diets. The limited success of the 5-a-day campaign since 2003 suggests that information measures alone are not enough to change behavior (62). Many countries are currently debating the introduction of a meat or carbon tax in order to reduce carbon footprints and improve population health (63). Evidence from the implementation of the UK Soft Drinks Industry Levy (64) indeed suggests that such targeted fiscal measures could be effective in steering the consumption of specific foods among consumers. Moreover, measures such as subsidies to reduce the cost of fruits and vegetables could be combined with improved access to these foods in retail environments in order to encourage consumption. This would be of particular importance to avoid placing a greater burden on the lowest-income households that already spend >15% of their total budget on food purchases (65). However, consumers’ motivation to increase intakes of fruit and vegetables is likely to also depend on several other factors, including knowledge and the extent to which proposed consumption pathways mirror habitual eating behaviors (66). School food policies and other procurement policies in public institutions that promote healthy and sustainable consumption are thus also warranted (67).

The current supply of fruit and vegetables in the United Kingdom is insufficient for meeting a hypothetical 5-a-day demand, something that other industrialized countries are likely to also be facing. In the United Kingdom, there is potential for local producers to close about one-third of this gap (A Wheeler, Food Foundation, personal communication, 2020). Horticulture is currently only ∼3% of United Kingdom's croppable area, of which the vast majority is dedicated to production of cereals and temporary grass for animal fodder (68). There is an opportunity to expand the UK horticulture sector, but this would require retraining of farmers, rebuilding of food supply chains, and tackling current problems of high input costs and low availability of agricultural workers (69) at harvest time. Similar to successful food policy programs elsewhere (70), the realization of our proposed pathways to 5-a-day at scale would necessitate collaboration and engagement across sectors and disciplines, involving stakeholders across policy, academia, civil society, and farming communities (27). However, in an environment where food systems are already rapidly evolving to cope with increasingly frequent climate and disease events, there may never be a better time to make bold changes in favor of healthy and sustainable diets.

In conclusion, there are multiple pathways to consumption of 5-a-day that would benefit both people's health and the environment, providing a range of policy options from which governments can select according to their priorities. Our results show that the pathways prioritizing vegetables over fruit and favoring an increased consumption of UK-produced varieties would achieve a better balance of benefits across health and reduction in GHGEs and water use. Achieving these dietary shifts at scale is likely to require a redesign of policy measures to ensure availability, affordability, and acceptability among both consumers and producers. The proposed dietary changes would not be sufficient to reach national and global sustainability targets; hence, additional measures to reduce the environmental footprints of UK diets are equally critical to consider.

## Supplementary Material

nqab076_Supplemental_Files

## Data Availability

Data described in the manuscript, code book, and analytic code will be made available upon request pending application and approval by the corresponding author.

## Notes

This study forms part of the Sustainable and Healthy Food Systems (SHEFS) project supported by the Wellcome Trust's Our Planet Our Health program (grant 205200/Z/6/Z; RG, PFDS, ADD, ON, and AT). The contribution by PEC was funded by the Swedish Research Council FORMAS (grant 2016-00353) and KI Foundations and Funds (grants 2019-01721 and 2019-02016). JM was funded by the Wellcome Trust project Complex Urban Systems for Sustainability and Health (CUSSH; grant 209387/Z/17/Z). The funders had no role in the study design, data analysis or writing, or the decision to submit for publication. All authors are independent from the funders and had full access to all data. All authors take responsibility for data integrity and accuracy of the analysis.

RG, PFDS, ADD, ON, and AT had financial support from the Wellcome Trust's Our Planet Our Health programme for the submitted work, PEC had financial support from the Swedish research council FORMAS and KI Foundations and Funds for the submitted work, JM had financial support from the Wellcome Trust project Complex Urban Systems for Sustainability and Health (CUSSH) for the submitted work,

Supplemental Figures 1–3, Supplemental Tables 1–12, and Supplemental Data 1 are available from the “Supplementary data” link in the online posting of the article and from the same link in the online table of contents at https://academic.oup.com/ajcn/.

Abbreviations used: CO_2_eq, carbon dioxide equivalents; GBD, Global Burden of Disease; GHGE, greenhouse gas emission; NCD, noncommunicable disease; NDNS, National Diet and Nutrition Survey; WF, water footprint; YLL, years of life lost.
