# Supplementary material for: Pathways to “5-a-day”: modeling the health impacts and environmental footprints of meeting the target for fruit and vegetable intake in the United Kingdom
Source: Am J Clin Nutr. 2021 Apr 19;114(2):530–9. doi: 10.1093/ajcn/nqab076 (PMC8326030; doi:10.1093/ajcn/nqab076)
Supplement: nqab076_Supplemental_Files [file nqab076_supplemental_files.zip › Online Supporting Material 20210203.docx]

Online Supplementary Material

Pathways to “5-a-day”: modeling the health impacts and environmental footprints of meeting the target for fruit and vegetable intake in the United Kingdom

[**1 MATERIAL AND METHODS** 4](#_Toc62736498)

[1.1 Dietary data 4](#_Toc62736499)

[**Supplementary Figure 1.** Participant flow according to selection criteria. 4](#_Toc62736500)

[**Supplementary Table 1.** Contribution of each survey year of the National Diet and Nutrition Survey (NDNS) to the total sample size (N=4,528). 5](#_Toc62736501)

[**Supplementary Figure 2.** Distribution of the reported dietary energy intake for all individuals in the sample (N=4,528). 5](#_Toc62736502)

[**Supplementary Table 2.** Reported dietary energy intake for all individuals in the sample as well as according to sex and age groups. 6](#_Toc62736503)

[**Supplementary Table 3.** Sub food groups disaggregated to provide a more detailed quantification of the reported intake of different varieties of fruit and vegetables 6](#_Toc62736504)

[**Supplementary Table 4.** New fruit and vegetable-types. 8](#_Toc62736505)

[1.2 Trade data, environmental footprints and cost of foods 9](#_Toc62736506)

[1.2.1 Determining environmental footprints of foods 9](#_Toc62736507)

[**Supplementary Table 5**. Main suppliers of livestock produce in the UK. 10](#_Toc62736508)

[1.2.2 Captions for Supplementary Data 1 (trade data, environmental footprints and cost of foods) 11](#_Toc62736509)

[1.3 Optimization 11](#_Toc62736510)

[1.3.1 Linear programming 11](#_Toc62736511)

[1.3.2 Optimized scenarios 13](#_Toc62736512)

[**Supplementary Table 6.** Names and characteristics of all modeled scenarios. 13](#_Toc62736513)

[**Supplementary Table 7.** List of fruit and vegetable-types suitable for increased production in the UK. 14](#_Toc62736514)

[1.4 Health impact modeling 14](#_Toc62736515)

[**Supplementary Table 8**. Underlying cause of death classifications (ICD-10) used for each health outcome. 14](#_Toc62736516)

[**2 RESULTS** 16](#_Toc62736517)

[2.1 Optimization 16](#_Toc62736518)

[**Supplementary Table 9.** Changes in key parameters (food groups, environmental impacts and cost) for the optimized scenarios compared to the average intake and its 95% Confidence Interval (CI). 16](#_Toc62736519)

[**Supplementary Figure 2.** Greenhouse gas emissions (g) and water footprint (liters) of only fruit and vegetables in the four optimized pathways. 18](#_Toc62736520)

[2.2 Health impact modeling 19](#_Toc62736521)

[**Supplementary Table 10**. Years of life gained (million) for each outcome over 20 years for males and females combined, including upper and lower health impact estimates (95% confidence intervals). 19](#_Toc62736522)

[**Supplementary Table 11.** Years of life gained (million) for each outcome over 30 years for males and females combined, including upper and lower health impact estimates (95% confidence intervals). 20](#_Toc62736523)

[**Supplementary Table 12.** Changes in life expectancy at birth, including upper and lower health impact estimates (95% confidence intervals). 21](#_Toc62736524)

[**3 References** 22](#_Toc62736525)

**Other Supplementary Material for this manuscript includes:**

Supplementary Data 1 (separate file)

# **1 MATERIAL AND METHODS**

## 1.1 Dietary data

This study used dietary data from United Kingdom (UK) National Diet and Nutrition Survey (NDNS), waves 5-9 (2012/13-2016/17). This is a is a rolling program of cross-sectional surveys based on a 4-day food diary, which provides amounts (in grams) of everything eaten or drunk over four consecutive days, per main food group (highest level of food aggregation), sub food group (lower level of food aggregation) and per individual (discrete) food item. (1). For this analysis, dietary data based on 18,006 food diaries from 4,528 individuals aged 12-95 years and reported over 3 or 4 days were used (Supplementary Figure 1). Approximately 2.4% (n=107) of the individuals included in our analyses reported dietary information for 3 days only, while the rest provided 4 days of dietary information. Each survey-year contributed by around 20%, respectively, to the sum of participants (N=4528) for the five waves (Supplementary Table 1). The different survey-waves sampled different people.

### **Supplementary Figure 1.** Participant flow according to selection criteria.

### **Supplementary Table 1.** Contribution of each survey year of the National Diet and Nutrition Survey (NDNS) to the total sample size (N=4,528).

|  | | **Number of individuals** | **Percent** |
| --- | --- | --- | --- |
| NDNS Wave | 5 | 849 | 18.8 |
|  | 6 | 934 | 20.6 |
|  | 7 | 951 | 21.0 |
|  | 8 | 950 | 21.0 |
|  | 9 | 844 | 18.6 |
|  | Total | 4528 | 100.0 |
|  |  |  |  |

NDNS = National Diet and Nutrition Survey.

The mean dietary energy intake was 1744 kcal per day. The dietary energy is provided with the dietary data as provided by Public Health England’s Nutrient Databank (NDB), specially designed for the NDNS rolling program (2). Supplementary Figure 2 displays the distribution of the reported dietary energy intake for the sample dietary days (N=4,528) and Supplementary Table 2 shows the dietary energy intake for the total sample as well as according to sex and age groups (≥18, 19-64, 65+).


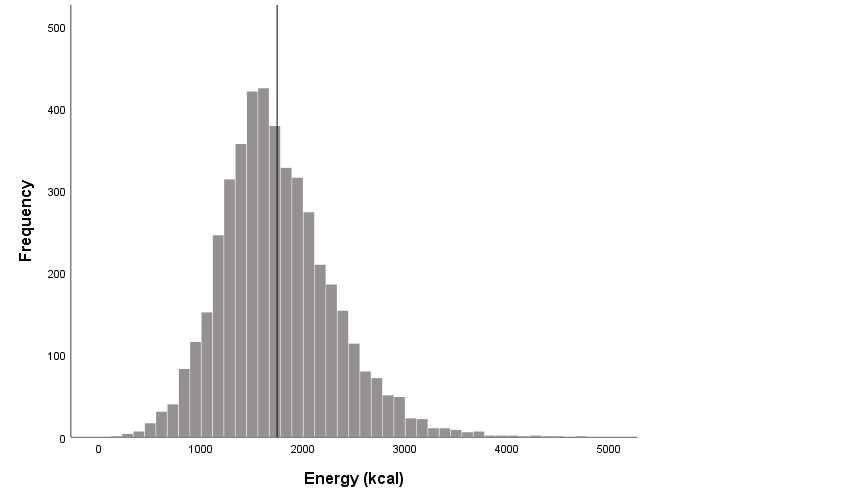


### **Supplementary Figure 2.** Distribution of the reported dietary energy intake for all individuals in the sample (N=4,528).

### **Supplementary Table 2.** Reported dietary energy intake for all individuals in the sample as well as according to sex and age groups.

|  | **All** | **Male** | | | **Female** | | |
| --- | --- | --- | --- | --- | --- | --- | --- |
| Age group | 12-95 years | 12 to 18 years | 19 to 64 years | 65+ years | 12 to 18 years | 19 to 64 years | 65+ years |
|  | **Kcal** | **Kcal** | **Kcal** | **Kcal** | **Kcal** | **Kcal** | **Kcal** |
| Mean | 1744 | 1901 | 2085 | 1839 | 1571 | 1592 | 1461 |
| 95% Confidence Interval | 1728, 1760 | 1857, 1945 | 2048, 2122 | 1789, 1889 | 1536, 1606 | 1568, 1617 | 1426, 1496 |
| Median | 1680 | 1829 | 2065 | 1796 | 1533 | 1563 | 1444 |
| Interquartile range | 691 | 683 | 795 | 574 | 531 | 603 | 515 |
| Min | 181 | 555 | 383 | 353 | 503 | 182 | 464 |
| Max | 4732 | 4554 | 4732 | 3589 | 3671 | 4088 | 3065 |

The method of aggregating individual (discrete) food items from waves 5-9 into main food groups and sub food groups, and the calculation of the energy content for each food entry within a main/sub food group, has been described elsewhere (3). In total, 169 sub food groups were identified. The seventeen sub food groups that described varieties of fruit and vegetables (Supplementary Table 3) were disaggregated (broken down) to 4336 discrete food items reported as consumed in order to enable a more detailed quantification of the intake of different types of fruit and vegetables. Breaking down the sub food groups into the discrete food items made it possible to distinguish different types of fruit and vegetables that were part of one and the same sub food group (e.g. 25 different types of vegetables were contained in the sub food group “Salad and other raw vegetables”). It also enabled the grouping of some fruit and vegetable-types (F&V-types) that were part of two different sub food groups (e.g. amount of the food item fresh celery in sub food group “Salad and other raw vegetables” and amount of the food item canned celery in sub food group “Other vegetables including homemade dishes” were grouped and labeled as “Celery”). As a result of this procedure, 112 F&V-types were distinguished and each of their corresponding amounts reported as consumed were grouped (Supplementary Table 4).

Total fruit and vegetable consumption considered all F&V-types consumed as discrete items (not in mixed/composite dishes) and included legumes (not peanuts or soya-based foods used as meat substitutes) but excluded fruit juices and potatoes. After the procedure of grouping fruit and vegetables according to the 112 distinguished types, the sub food group “Other vegetables including homemade dishes”, still contained a total of 4.2 grams (per day) of the abovementioned foods not being considered as F&V in this study. This residual amount was contained in the sub food group (re-labelled as “Other vegetables including homemade dishes – not part of veg”), which together with the remaining 152 (non-F&V) sub food groups and the new grouping of 112 F&V-types resulted in a total of 265 compositionally distinct food groups used to estimate the observed UK-diet and the optimized pathways (Supplementary Data 1, Sheets 4a-e). The fruit and vegetable food groups were not aggregated as broadly as the other food groups in order to provide a more detailed overview of the reported intakes of different varieties of fruit and vegetables and to facilitate comparisons between them in terms of sustainability.

### **Supplementary Table 3.** Sub food groups disaggregated to provide a more detailed quantification of the reported intake of different varieties of fruit and vegetables

| **Sub food group** | **Average grams per day** |
| --- | --- |
|  |  |
| \| Apples and pears not canned \| \| --- \| \| Baked beans \| \| Bananas \| \| Beans and pulses incl ready meal & homemade dishes \| \| Canned fruit in juice \| \| Canned fruit in syrup \| \| Carrots not raw \| \| Carrots raw \| \| Citrus fruit not canned \| \| Green beans not raw \| \| Leafy green vegetables not raw \| \| Other fruit not canned \| \| Other vegetables including homemade dishes \| \| Peas not raw \| \| Salad and other raw vegetables \| \| Tomatoes not raw \| \| Tomatoes raw \| | \| 23.9 \| \| --- \| \| 15.5 \| \| 23.8 \| \| 5.5 \| \| 1.1 \| \| 0.8 \| \| 11.6 \| \| 1.9 \| \| 11.8 \| \| 2.7 \| \| 12.1 \| \| 28.3 \| \| 41.8 \| \| 8.1 \| \| 20.2 \| \| 12.1 \| \| 12.8 \| |
|  |  |
|  |  |

### **Supplementary Table 4.** New fruit and vegetable-types.

| **Fruits (48 types)** | **Average grams per day^1^** | | **Vegetables (64 types)** | **Average grams per day^1^** |
| --- | --- | --- | --- | --- |
| Apples | 18.3 | Artichoke | | <0.1 |
| Apricots | 0.4 | Asparagus | | 0.9 |
| Bananas | 23.8 | Aubergine | | 0.6 |
| Bilberries | 0.3 | Avocado | | 1.2 |
| Blackberries | 0.4 | Baked beans | | 15.5 |
| Blackcurrants | 0.1 | Bamboo shoots | | <0.1 |
| Blueberries | 1.4 | Bean sprouts | | 0.3 |
| Breadfruit | <0.1 | Bean and lentil-based foods | | 1.1 |
| Canned fruit | 1.9 | Beetroot | | 1.2 |
| Cherries | 0.3 | Broad beans | | 0.4 |
| Cranberries | 0.1 | Broccoli | | 6.6 |
| Currants | <0.1 | Brussels sprouts | | 1.0 |
| Dates | 0.4 | Butternut squash | | 0.9 |
| Figs | 0.2 | Cabbage | | 3.7 |
| Gojiberries | <0.1 | Carrots | | 13.6 |
| Gooseberries | <0.1 | Cassava | | <0.1 |
| Grapefruit | 0.8 | Cauliflower | | 2.9 |
| Grapes | 5.1 | Celeriac | | 0.1 |
| Guava | <0.1 | Celery | | 1.0 |
| Kiwi | 0.9 | Chicory | | <0.1 |
| Lemons | 0.2 | Chinese leaves | | <0.1 |
| Limes | 0.1 | Cho-Cho | | <0.1 |
| Loganberries | <0.1 | Courgettes | | 1.0 |
| Lychees | <0.1 | Cucumber | | 4.8 |
| Mangoes | 1.4 | Endive | | <0.1 |
| Melons | 1.8 | Fennel | | 0.1 |
| Mixed fruits | 0.6 | Fresh herbs | | 0.2 |
| Nectarines | 1.3 | Garlic | | 0.3 |
| Oranges | 5.3 | Ginger | | 0.2 |
| Papaya | 0.1 | Gourd | | <0.1 |
| Passion fruit | <0.1 | Green beans | | 2.7 |
| Peaches | 1.1 | Horseradish | | <0.1 |
| Pears | 5.6 | Kohl rabi | | <0.1 |
| Physalis | <0.1 | Leeks | | 1.4 |
| Pineapple | 1.6 | Lentils | | 1.5 |
| Platain | 0.1 | Lettuce | | 4.1 |
| Plums | 1.2 | Marrow | | <0.1 |
| Pomegranate | 0.1 | Mixed leaf salad | | 1.4 |
| Prunes | 0.2 | Mixed vegetables | | 0.6 |
| Quinces | <0.1 | Mushrooms | | 4.6 |
| Raspberries | 1.2 | Mustard cress | | <0.1 |
| Red currants | <0.1 | Okra | | 0.1 |
| Rhubarb | 0.2 | Olives | | 0.6 |
| Sharon fruit | 0.2 | Onions | | 13.9 |
| Strawberries | 4.7 | Other beans | | 2.5 |
| Tamarinds | <0.1 | Pak choi | | 0.1 |
| Tangerines | 5.3 | Parsnips | | 1.5 |
| Watermelon | 0.9 | Peas | | 8.2 |
|  |  | Peppers | | 5.9 |
|  |  | Pumpkin | | <0.1 |
|  |  | Raddicio | | <0.1 |
|  |  | Radish | | 0.2 |
|  |  | Rocket | | 0.4 |
|  |  | Spinach | | 1.5 |
|  |  | Swede | | 1.0 |
|  |  | Sweet potato | | 1.6 |
|  |  | Sweetcorn | | 2.5 |
|  |  | Swiss chard | | <0.1 |
|  |  | Tomatoes not raw | | 12.1 |
|  |  | Tomatoes raw | | 12.8 |
|  |  | Turnips | | 0.4 |
|  |  | Water chestnut | | <0.1 |
|  |  | Watercress | | 0.1 |
|  |  | Yams | | 0.1 |

^1^Confidence interval for total fruit and vegetable consumption, respectively, is found in Supplementary Table 9.

An average energy content for each of the 112 distinguished F&V-types per 100 g was established by calculating the total energy content (as provided by the NDNS data) for each original individual F&V item (e.g. for fresh apples, dried apples, baked apples and stewed apples) being part of the new F&V-type, summing these into a grand total (total kcal), dividing this grand total by the total intake of the new F&V-type (g), and multiplying this by a 100 (to get the energy content per 100 grams).

## 1.2 Trade data, environmental footprints and cost of foods

### 1.2.1 Determining environmental footprints of foods

Estimates of greenhouse gas emissions (GHGE) and water footprints (WF) associated with each of the 112 new F&V-types and the 153 sub food groups representing the remaining dietary intake (Supplementary Data 1) were calculated using a Life Cycle Inventory (LCI) compiled from the relevant literature in the UK and Europe (Supplementary Data 1). For sub food groups or F&V-types where specific emissions estimates were not available in the literature, representative items for a sub food group or F&V-type for which emissions data were available were used as a reference point for all foods within the sub group or F&V-type. In some cases (certain fruits, vegetables, as well as organ meat), an average for compositionally distinct sub food groups or F&V-types was used.

The below information describes further how information from relevant literature on trade and environmental footprints data (Supplementary Data 1) were used to determine the environmental impact of sub food groups or F&V-types:

**1. Animal products:**

1. Countries (including the UK) that supplied at least 10% of the total amount of the food available in the UK were identified. The rest of the supply was categorized as “Supply Global” (Supplementary Table 5).
2. Each identified country (and sub food group) was assigned its specific GHGE and WF if possible.
3. Environmental footprints for beef were adjusted to reflect the expected amount of beef coming from bovine cows (87%) and milk cows (13%), respectively (*Herrero 2020*, from personal communication).
4. The UK specific values, country specific values for imported foods, and the global mean value (for “Supply global”) were used to create weighted environmental footprints (Supplementary Data 1, sheet 2a) for the UK and for imported goods based on their respective contribution to food availability (Supplementary Data 1, sheet 1a).

### **Supplementary Table 5**. Main suppliers of livestock produce in the UK.

| **Food** | **Country** | **% of total supply** | **Carbon dioxide equivalents /kg of product** | **Blue water footprint/kg of product** |
| --- | --- | --- | --- | --- |
|  |  |  |  |  |
| PIG MEAT | Denmark | 13.2 | 11.0 | 1030 |
|  | Germany | 10.7 | 11.0 | 1030 |
|  | UK | 49.8 | 11.0 | 1030 |
|  | Supply Global | 26.3 | 12.3 | 1796 |
| MILK | UK | 74.4 | 2.2 | 24 |
|  | Global | 25.6 | 3.2 | 628 |
| EGGS | UK | 86.4 | 5.1 | 39 |
|  | Supply Global | 13.6 | 4.7 | 578 |
| BEEF AND BUFFALO | Ireland | 16.2 | 47.2 | 205 |
|  | UK | 72.3 | 47.2 | 205 |
|  | Supply Global | 11.5 | 90.7 | 1620 |
| SHEEP AND GOAT | New Zealand | 18.7 | 39.7 | 1803 |
|  | UK | 74.8 | 43.0 | 228 |
|  | Supply Global | 6.5 | 39.7 | 1803 |
| POULTRY | UK | 71.9 | 9.23 | 77 |
|  | Supply Global | 28.1 | 9.9 | 660 |
| OFFALS | Ireland | 12.3 | 25.7 | 544 |
|  | UK | 74.2 | 25.7 | 544 |
|  | Supply Global | 13.5 | 38.1 | 1470 |

**2. Fruit and vegetables:**

1. Environmental footprints for UK specific and imported F&V types were assigned (Supplementary Data 1). Since specific environmental footprints for fresh, frozen, dried, and canned produce were available for many fruit and vegetables (4,5), these footprints were thus first adjusted so that they corresponded to the proportions of fresh, frozen, dried, and canned produce as reported in the dietary data
2. The UK specific vs. Imported values were used to create weighted environmental footprints (Supplementary Data 1, sheet 2a) in accordance with the proportion of each food group/food being imported vs. locally produced (Supplementary Data 1, sheet 1a).

**3. Other foods**

Environmental footprints for UK produced and Imported foods were derived from literature (Supplementary Data 1).

1. Environmental footprints for UK specific and Imported foods were assigned.
2. The UK specific vs. Imported values were used to create weighted environmental footprints (Supplementary Data 1, sheet 2a) in accordance with the proportion of each food group/food being imported vs. locally produced (Supplementary Data 1, sheet 1a)

**4. Environmental footprints used for the different scenarios**

1. For the F&V-ALL and VEG-ALL scenarios, environmental footprints were used that were weighted in line with the proportion of domestic versus imported produce (Supplementary Data 1, sheet 1a)
2. For F&V-UK and VEG-UK scenarios, UK-specific environmental footprints were used for the fruit and vegetables being modified while the weighted values were used for all other foods (Supplementary Data 1, sheet 2a).

### 1.2.2 Captions for Supplementary Data 1 (trade data, environmental footprints and cost of foods)

**Supplementary Data 1.** Data in spreadsheet format (separate file). This file contains the trade and environmental footprint data used as well as the references upon which these were based. The file also contains details on the average price data used.

## 1.3 Optimization

### 1.3.1 Linear programming

Linear programming (LP) is the application of an algorithm for maximizing or minimizing a given linear objective function (goal function, the variable to be optimized) subjected to a set of linear constraints (conditions to be met) on a list of decision variables (6). A feasible solution is found when all conditions can be met. The minimization of the total relative deviation (TRD), which is the sum of all absolute (non-negative) values of relative deviations (RDs) as described in the main manuscript, from the current UK diet was chosen as the objective function of the LP model (7,8). This was applied to provide a proxy for the diet with the highest achievable similarity to the observed UK diet (1). LP was performed with the CBC (COIN-OR Branch Solver algorithm, which is part of the Excel® 2016 software add-in OpenSolver, V. 2.9.0 (9).

All solutions were isocaloric and contained the mean daily energy intake reported by the total study participants of the NDNS (Table S2). Average intakes (in grams) of the 153 sub food groups and 112 F&V-types were calculated by dividing the total intake for each sub food group and F&V-type, respectively, by the number of food diaries (18,006). These average intakes were calculated for modeling purposes and represented the observed food consumption in the UK. To provide a range for plausible sub food group and F&V-type intakes, confidence intervals (CI) for the average intakes were also quantified.

As a proxy for the similarity to the observed food intake, for each sub food group or F&V-type, the relative deviation (RD) of the modified diet from the average amount recorded for each sub food group or F&V-type in the NDNS (1) was calculated (Formula 1).

${RD}_{i} = \frac{m_{i}-M_{i}}{M_{i}}$ Formula 1

In Formula 1, *m* indicates the modified weight of the *i*-th sub food group or F&V-type in grams in the altered diet and *M_i_* is the consumed weight of the same sub food group or F&V-type as reported in the NDNS. For each scenario, the total RD (TRD) was minimized with linear optimization and all modeled scenarios were constrained to be isocaloric to the observed average UK-diet (1.744 kcal) (7,8). The TRD from all N (265) sub food groups and F&V-types in the model was calculated as the total sum of the absolute non-negative values of the RDs:

| $TRD = \sum_{i=1}^{N} abs(\mathrm{RD}_{i})$ | (2) |
| --- | --- |

In order to implement absolute values into the linear programming process, the non-negative values of RD were calculated as described before (7,8):

| abs(RD)_i_ ≥ (m_i_ − M_i_)/m_i_ and abs(RD)_i_ ≥ −(m_i_ − M_i_)/m_i_ | (3) |
| --- | --- |
|  |  |

Thus, for each standardized difference, its absolute (positive) value was calculated since the absolute RD (abs(RD))_i_, by definition, has to be greater than or equal to both the relative difference and its negative value.

The average relative deviation (ARD) from the observed food consumption was calculated to provide an average of the absolute RD of all (265) sub food groups and F&V-types, and was calculated by dividing the TRD by the total number of sub groups and F&V-types included in the model (N=265), as given in Formula 4:

| $ARD=TRD/N$ | (4) |
| --- | --- |

### 1.3.2 Optimized scenarios

We developed four possible pathways to achieving the 5-a-day recommendation, based on increasing consumption of F&V-types likely to be more sustainable and/or increase resilience of the UK food supply. Supplementary Table 6 maps the acronyms and characteristics of all optimized scenarios, including applied constraints (targets).

### **Supplementary Table 6.** Names and characteristics of all modeled scenarios.

| **Scenario and acronym** | **Target consumption of fruits** | **Target consumption of vegetables** | **Substitutions** |
| --- | --- | --- | --- |
| **1. F&V-ALL** | 400 g F&V – increase in F&V proportional to current intakes of both fruit and vegetables | | a) Kcal increase in fruit (**39.2 kcal**) substituting the same amount of kcal from sweet snacks  b) Kcal increase in vegetables (**41.0 kcal**) substituting the same amount of kcal from meat (including poultry) maintaining the same proportion of red/white meat |
| **2. VEG-ALL** | Equal to observed amount of fruits | Equal to the difference between F&V target of 400 g and the observed amount of fruits - Increase proportional to current intakes of vegetables | a) Kcal increase in vegetables (**66.7 kcal**) substituting the same amount of kcal from meat (including poultry) |
| **3. F&V-UK** | Total of 400 g – observed amounts of non-“UK-capable” F&V kept constant, increase only from “UK-capable” F&V | | a) Kcal increase in fruit (**20.4 kcal**) substituting the same amount of kcal from sweet snacks  b) Kcal increase in vegetables (**37.6**) substituting the same amount of kcal from meat (including poultry) |
| **4. VEG-UK** | Equal to observed amount of fruits | Equal to the difference between F&V target of 400 g and the observed amount of fruits - Increase only from “UK-capable” vegetables | a) Kcal increase in vegetables (**51.2 kcal**) substituting the same amount of kcal from meat (including poultry) |

The third and fourth scenario (F&V-UK and VEG-UK) were also modeled as per the F&V-ALL scenario, yet here, all additional consumption was constrained to be provided by only “UK-capable” fruit and/or vegetables from a list of F&V-types which are already produced in the UK and identified as a crop that could be expanded (Supplementary Table 7).

### **Supplementary Table 7.** List of fruit and vegetable-types suitable for increased production in the UK.

| **Fruits** | **Vegetables** |
| --- | --- |
| Apples | Onions |
| Cherries | Carrots |
| Plums | Broccoli |
| Raspberries | Peppers |
| Strawberries | Cucumber |
|  | Broad beans |
|  | Cabbage |
|  | Cauliflower |
|  | Green beans |
|  | Leeks |
|  | Brussels sprouts |
|  | Asparagus |
|  | Lettuce |
|  | Peas |
|  | Courgettes |
|  |  |

## 1.4 Health impact modeling

Health impact calculations were carried out on the basis of a previously applied strategy (3), using the life table model IOMLIFET (10), implemented in R (11).

The IOMLIFET model estimates survival patterns in the population over time based on age-specific mortality rates. Based on information of the change in risk-exposure and the exposure-response function, the health impact assessment is calculated by adjusting the underlying mortality rates and comparing the impacted life table to the baseline life table. Supplementary Table 8 maps the modeled health outcomes to International Classification of Diseases (ICD10) codes (12), which are needed for the disease-specific mortality figures applied in the model.

###

### **Supplementary Table 8**. Underlying cause of death classifications (ICD-10) used for each health outcome.

|  |  |  |
| --- | --- | --- |
| **Health outcome** | **ICD-10 underlying cause of death classification** |  |
| Ischemic heart disease | I20 – I25.9 |  |
| Ischemic stroke | G45-G46.8, I60-I63.9, I65-I66.9, I67.0-I67.3, I67.5-I67.6, I68.1-I68.2, I69.0-I69.3 |  |
| Tracheal, bronchus, and lung cancer | C33-C34.9, D02.1-D02.3, D14.2-D14.3, D38.1 |  |
| Esophageal cancer | C15-C15.9, D00.1, D13.0 |  |
| Diabetes mellitus type 2 | E11-E11.1, E11.3-E11.9 |  |
| Colorectal cancer | C18-C21.9, D01.0-D01.3, D12-D12.9, D37.3-D37.5 |  |

ICD = International Classification of Diseases

Age-specific and sex-specific population size estimates from the Office for National Statistics (13), as well as data on all-cause mortality and disease-specific mortality from the Global Burden of Disease (GBD results-tool (14) were combined to create input data for the UK. The disease-specific mortality data were only available by 5-year age ranges and not in single-year-of-age format. Therefore, single-year-of-age estimates were generated from the age-grouped data by monotonic cubic spline interpolation as suggested in literature [10]. Life tables were created separately for each outcome in order to isolate the impact due to each outcome alone.

Evidence-based dose-response relationships (relative risks) between dietary intake and mortality from chronic diseases were obtained from the latest GBD study (15). The relative risks from GBD which were expressed in terms of a harmful risk factor (e.g. "diet low in fruit") were inverted to create relative risks for a positive change in diet. GBD provides relative risks for females and males combined, by 5-year age ranges, starting from the age of 25. The relative risks for each risk, outcome, and 5-year age range were weighted according to the age distribution in the UK population to create one single relative risk for each risk-outcome pair. Ages below 25 were assigned the same relative risk as the 25-29 age group, although the baseline mortality from the included dietary risks was almost non-existent in the UK population <25.

Changes in life expectancy at birth were calculated as the difference between baseline life expectancy (the expected life years divided by the starting population) and the impacted (modeled) life expectancy (the impacted expected life years by the impacted starting population).

# **2 RESULTS**

## 2.1 Optimization

### **Supplementary Table 9.** Changes in key parameters (food groups, environmental impacts and cost) for the optimized scenarios compared to the average intake and its 95% Confidence Interval (CI).

| **Output** | **Unit** | **Average intake** | | | | | **Lower CI** | | | | | **Upper CI** | | | | |
| --- | --- | --- | --- | --- | --- | --- | --- | --- | --- | --- | --- | --- | --- | --- | --- | --- |
|  |  | Observed UK diet | F&V-ALL | VEG-ALL | F&V-UK | VEG-UK | Observed UK diet | F&V-ALL | VEG-ALL | F&V-UK | VEG-UK | Observed UK diet | F&V-ALL | VEG-ALL | F&V-UK | VEG-UK |
| Fruit and vegetables | | | | | | | | | | | | | | | | |
| Fruits^1^ | g | 88 | 154 | 88 | 134 | 88 | 87 | 154 | 87 | 133 | 87 | 89 | 154 | 89 | 134 | 89 |
|  | change (g) | na | 66 | 0 | 46 | 0 | na | 67 | 0 | 46 | 0 | na | 65 | 0 | 45 | 0 |
|  | change (%) | na | 76 | 0 | 52 | 0 | na | 78 | 0 | 53 | 0 | na | 74 | 0 | 51 | 0 |
| Vegetables (legumes)^1^ | g | 140 (32) | 246 (56) | 312 (72) | 266 (52) | 312 (59) | 138 (32) | 246 (57) | 313 (72) | 267 (52) | 313 (60) | 141 (32) | 246 (57) | 311 (72) | 266 (52) | 311 (59) |
|  | change (g) | na | 106 (24) | 172 (40) | 126 (20) | 172 (27) | na | 108 (25) | 175 (40) | 129 (20) | 175 (28) | na | 105 (25) | 170 (40) | 125 (20) | 170 (27) |
|  | change (%) | na | 76 (75) | 123 (125) | 90 (63) | 123(84) | na | 78 (78) | 127 (125) | 93 (63) | 127 (88) | na | 74 (78) | 121 (125) | 89 (63) | 121 (84) |
| Substitutions | | | | | | | | | | | | | | | | |
| Sweet snacks^1^ | g | 49 | 39 | 49 | 44 | 49 | 48 | 38 | 48 | 43 | 48 | 49 | 39 | 49 | 44 | 49 |
|  | change (g) | na | -10 | 0 | -5 | 0 | na | -10 | 0 | -5 | 0 | na | -10 | 0 | -5 | 0 |
|  | change (%) | na | -20 | 0 | -10 | 0 | na | -21 | 0 | -10 | 0 | na | -20 | 0 | -10 | 0 |
| Red meat | g | 31 | 25 | 21 | 26 | 24 | 30 | 25 | 21 | 25 | 23 | 31 | 25 | 22 | 26 | 24 |
|  | change (g) | na | -6 | -10 | -5 | -7 | na | -5 | -9 | -5 | -7 | na | -6 | -9 | -5 | -7 |
|  | change (%) | na | -19 | -32 | -16 | -23 | na | -19 | -31 | -17 | -24 | na | -18 | -30 | -17 | -23 |
| Poultry meat | g | 41 | 33 | 29 | 34 | 32 | 41 | 33 | 28 | 34 | 31 | 42 | 34 | 29 | 35 | 32 |
|  | change (g) | na | -8 | -12 | -7 | -9 | na | -8 | -13 | -7 | -10 | na | -8 | -12 | -7 | -9 |
|  | change (%) | na | -20 | -29 | -17 | -22 | na | -19 | -31 | -17 | -24 | na | -18 | -30 | -17 | -23 |
| Processed meat | g | 43.86 | 35.63 | 30.54 | 36.41 | 33.73 | 43 | 35 | 30 | 36 | 33 | 44 | 36 | 31 | 37 | 34 |
|  | change (g) | na | -8 | -13 | -7 | -10 | na | -8 | -13 | -7 | -10 | na | -8 | -13 | -7 | -10 |
|  | change (%) | na | -19 | -30 | -17 | -23 | na | -19 | -31 | -17 | -24 | na | -18 | -30 | -17 | -23 |
| Environmental footprints | | | | | | | | | | | | | | | | |
| CO_2eq_./person/day | kg | 6.2 | 5.9 | 5.7 | 5.9 | 5.7 | 6.1 | 5.9 | 5.6 | 5.8 | 5.7 | 6.2 | 6.0 | 5.7 | 5.9 | 5.8 |
|  | change (kg) | na | -0.3 | -0.5 | -0.3 | -0.4 | na | -0.2 | -0.5 | -0.3 | -0.4 | na | -0.2 | -0.5 | -0.3 | -0.4 |
|  | change (%) | na | -4.1 | -8.2 | -4.8 | -7.0 | na | -4.0 | -8.2 | -4.9 | -5.9 | na | -3.6 | -8.1 | -4.8 | -6.5 |
| CO_2eq_./UK population/year^2^ | Mt | 147.9 | 141.8 | 135.7 | 140.8 | 137.5 | 146.2 | 140.3 | 134.2 | 139.0 | 137.5 | 148.6 | 143.2 | 136.6 | 141.4 | 139.0 |
|  | change (Mt) | na | -6.1 | -12.2 | -7.1 | -10.4 | na | -5.9 | -12.0 | -7.2 | -8.7 | na | -5.4 | -12.0 | -7.2 | -9.6 |
| WF/person/day | L | 611.4 | 614.2 | 610.5 | 605.6 | 605.4 | 605.7 | 608.5 | 604.7 | 60<0.1 | 599.5 | 616.9 | 619.6 | 616.0 | 611.2 | 610.9 |
|  | change (L) | na | 2.8 | -0.9 | -5.8 | -6.0 | na | 2.8 | -1.0 | -5.7 | -6.2 | na | 2.7 | -0.9 | -5.7 | -6.0 |
|  | change (%) | na | 0.5 | -0.2 | -0.9 | -1.0 | na | 0.5 | -0.2 | -0.9 | -1.0 | na | 0.4 | -0.1 | -0.9 | -1.0 |
| WF/UK population/year^2^ | km^3^ | 14.7 | 14.7 | 14.6 | 14.5 | 14.5 | 14.5 | 14.6 | 14.5 | 14.4 | 14.4 | 14.8 | 14.8 | 14.8 | 14.6 | 14.6 |
|  | change (km^3^) | na | <0.17 | -<0.12 | -0.14 | -0.14 | na | <0.17 | -<0.12 | -0.14 | -0.15 | na | <0.16 | -<0.12 | -0.14 | -0.14 |
| Cost | | | | | | | | | | | | | | | | |
| Cost | GBP | 6.78 | 7.14 | 7.12 | 7.24 | 7.21 | 6.7 | 7.1 | 7.1 | 7.2 | 7.1 | 6.8 | 7.2 | 7.2 | 7.3 | 7.3 |
|  | change (GBP) | na | 0.36 | 0.34 | 0.46 | 0.43 | na | 0.4 | 0.4 | 0.5 | 0.4 | na | 0.4 | 0.4 | 0.5 | 0.5 |
|  | change (%) | na | 4.4 | 4.4 | 5.9 | 5.9 | na | 6.0 | 6.0 | 7.5 | 6.0 | na | 5.9 | 5.9 | 7.4 | 7.4 |

CO_2_eq = Carbo dioxide equivalents.

GBP = Great Britain Pound.

na = not applicable.

WF = Water footprint.

^1^Per day and person.

^2^Per year for UK population of 65.65 million inhabitants.


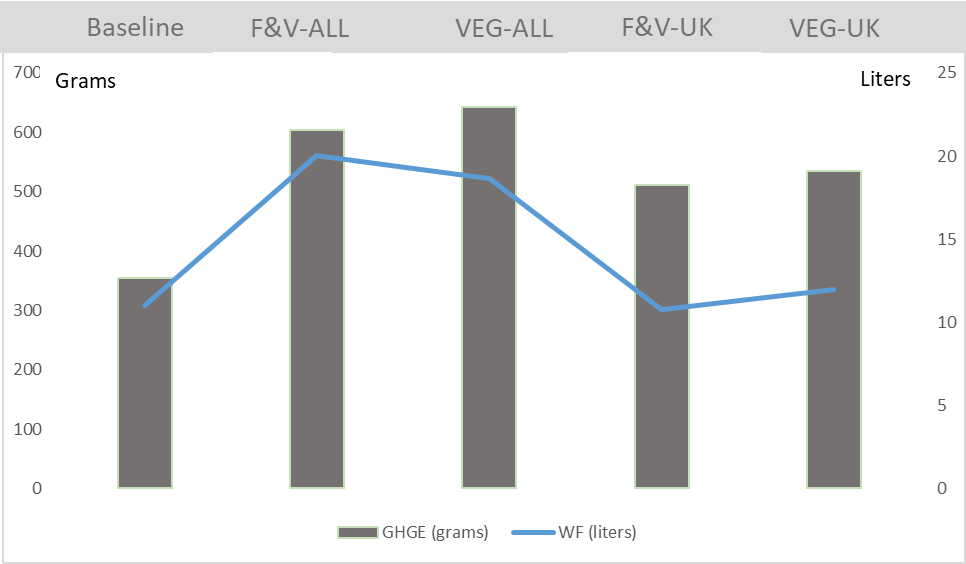


### **Supplementary Figure 3.** Greenhouse gas emissions (g) and water footprint (liters) of only fruit and vegetables in the four optimized pathways.

## 2.2 Health impact modeling

### **Supplementary Table 10**. Years of life gained (million) for each outcome over 20 years for males and females combined, including upper and lower health impact estimates (95% confidence intervals).

|  | **Health outcome** | **Years of life gained** | | | |
| --- | --- | --- | --- | --- | --- |
|  |  | **F&V-ALL** | **VEG-ALL** | **F&V-UK** | **VEG-UK** |
| Central estimate for RR | Ischemic heart disease | 2 821 326 | 3 406 093 | 2 693 869 | 2 990 039 |
|  | Ischemic stroke | 617 969 | 314 408 | 549 107 | 342 491 |
|  | Esophageal cancer | 6 973 | 0 | 4 927 | 0 |
|  | Tracheal. Bronchus, and lung cancer | 17 321 | 0 | 12 159 | 0 |
|  | Colorectal cancer | 6 338 | 10 220 | 5 633 | 7 669 |
|  | Diabetes mellitus | 58 021 | 58 710 | 48 323 | 45 074 |
|  | **Total** | **3 527 947** | **3 789 430** | **3 314 018** | **3 385 273** |
| Lower 95% CI for RR | Ischemic heart disease | 1 042 551 | 1 289 240 | 1 007 602 | 1 130 926 |
|  | Ischemic stroke | 309 279 | 73 546 | 245 550 | 80 638 |
|  | Esophageal cancer | 1 580 | 0 | 1 105 | 0 |
|  | Tracheal. Bronchus, and lung cancer | 7 370 | 0 | 5 153 | 0 |
|  | Colorectal cancer | 3 100 | 5 006 | 2 785 | 3 787 |
|  | Diabetes mellitus | 22 292 | 27 920 | 19 186 | 21 358 |
|  | **Total** | **1 386 173** | **1 395 712** | **1 281 380** | **1 236 710** |
| Upper 95% CI for RR | Ischemic heart disease | 4 026 778 | 4 745 693 | 3 881 011 | 4 264 125 |
|  | Ischemic stroke | 838 219 | 501 953 | 769 380 | 543 766 |
|  | Esophageal cancer | 12 009 | 0 | 8 576 | 0 |
|  | Tracheal. Bronchus, and lung cancer | 27 419 | 0 | 19 331 | 0 |
|  | Colorectal cancer | 9 690 | 15 552 | 8 591 | 11 674 |
|  | Diabetes mellitus | 87 936 | 81 181 | 72 753 | 62 724 |
|  | **Total** | **5 002 052** | **5 344 378** | **4 759 641** | **4 882 288** |

CI = Confidence interval.

RR=relative risk.

|  |  |  | | | |
| --- | --- | --- | --- | --- | --- |
|  | **Health outcome** | **Years of life gained** | | | |
|  |  | **F&V-ALL** | **VEG-ALL** | **F&V-UK** | **VEG-UK** |
| Central estimate for RR | Ischemic heart disease | 6 398 931 | 7 733 044 | 6 108 510 | 6 783 559 |
|  | Ischemic stroke | 1 333 645 | 677 939 | 1 184 800 | 738 551 |
|  | Esophageal cancer | 63 750 | 0 | 45 044 | 0 |
|  | Tracheal. Bronchus, and lung cancer | 159 874 | 0 | 112 217 | 0 |
|  | Colorectal cancer | 56 884 | 91 737 | 50 559 | 68 833 |
|  | Diabetes mellitus | 129 944 | 131 486 | 108 223 | 100 945 |
|  | **Total** | **8 143 029** | **8 634 206** | **7 609 354** | **7 691 888** |
| Lower 95% CI for RR | Ischemic heart disease | 2 357 426 | 2 916 450 | 2 278 264 | 2 557 639 |
|  | Ischemic stroke | 666 871 | 158 474 | 529 360 | 173 760 |
|  | Esophageal cancer | 14 446 | 0 | 10 099 | 0 |
|  | Tracheal. Bronchus, and lung cancer | 68 017 | 0 | 47 547 | 0 |
|  | Colorectal cancer | 27 821 | 44 929 | 24 995 | 33 991 |
|  | Diabetes mellitus | 49 922 | 62 526 | 42 966 | 47 831 |
|  | **Total** | **3 184 502** | **3 182 379** | **2 933 232** | **2 813 221** |
| Upper 95% CI for RR | Ischemic heart disease | 9 152 152 | 10 799 836 | 8 818 591 | 9 695 650 |
|  | Ischemic stroke | 1 810 118 | 1 082 909 | 1 661 132 | 1 173 257 |
|  | Esophageal cancer | 109 814 | 0 | 78 409 | 0 |
|  | Tracheal. Bronchus, and lung cancer | 253 135 | 0 | 178 433 | 0 |
|  | Colorectal cancer | 86 980 | 139 619 | 77 116 | 104 795 |
|  | Diabetes mellitus | 196 953 | 181 820 | 162 942 | 140 477 |
|  | **Total** | **11 609 151** | **12 204 185** | **10 976 622** | **11 114 179** |

### **Supplementary Table 11.** Years of life gained (million) for each outcome over 30 years for males and females combined, including upper and lower health impact estimates (95% confidence intervals).

CI = Confidence interval.

RR = relative risk.

### **Supplementary Table 12.** Changes in life expectancy at birth, including upper and lower health impact estimates (95% confidence intervals).

| **Diet impacts^1^** | **Units** | **Current diet** | **F&V-ALL** | **VEG-ALL** | **F&V-UK** | **VEG-UK** |
| --- | --- | --- | --- | --- | --- | --- |
| Life expectancy | Years (months) | 81.1 (973)^2^ | 81.3 (976) | 81.3 (976) | 81.3 (976) | 81.3 (976) |
|  | Change in months - Lower 95% CI for RR | na | 3.1 | 3.0 | 2.8 | 2.6 |
|  | Years (months) | 81.1 (973)^2^ | 82.0 (985) | 82.1 (985) | 82.0 (984) | 82.0 (984) |
|  | Change in months - Upper 95% CI for RR | na | 11.5 | 11.6 | 10.7 | 10.5 |

RR = relative risk.

^1^ Assessed using life table models.

^2^Average life expectancy of the basline population (81.07. years).

# **3 References**

1. National Diet and Nutrition Survey [Internet]. GOV.UK. [cited 2020 Mar 1]. Available from: https://www.gov.uk/government/collections/national-diet-and-nutrition-survey

2. Appendix A_Dietary data collection and editing for NDNS RP_Updated for Y7-8 NDNS. Results from Years 7-8 (combined) of the Rolling Programme (2014/15 – 2015/16): UK.

3. Milner J, Green R, Dangour AD, Haines A, Chalabi Z, Spadaro J, Markandya A, Wilkinson P. Health effects of adopting low greenhouse gas emission diets in the UK. BMJ Open. 2015;5:e007364.

4. Frankowska A, Jeswani HK, Azapagic A. Environmental impacts of vegetables consumption in the UK. Sci Total Environ. 2019;682:80–105.

5. Frankowska A, Jeswani HK, Azapagic A. Life cycle environmental impacts of fruits consumption in the UK. J Environ Manage. 2019;248:109111.

6. Dantzig GB. 1947. Maximization of a linear function of variables subject to linear inequality. Koopmans TC, editor Activity analysis of production and allocation. New York-London: Wiley & Chapman-Hall; 1951. p. 339–47.

7. Darmon N, Ferguson EL, Briend A. A Cost Constraint Alone Has Adverse Effects on Food Selection and Nutrient Density: An Analysis of Human Diets by Linear Programming. J Nutr. 2002;132:3764–71.

8. Eustachio Colombo P, Patterson E, Schäfer Elinder L, Lindroos AK, Sonesson U, Darmon N, Parlesak A. Optimizing School Food Supply: Integrating Environmental, Health, Economic, and Cultural Dimensions of Diet Sustainability with Linear Programming. Int J Environ Res Public Health. 2019;16:3019.

9. Mason AJ. OpenSolver - An Open Source Add-in to Solve Linear and Integer Progammes in Excel. In: Klatte D, Lüthi H-J, Schmedders K, editors. Operations Research Proceedings 2011 [Internet]. Berlin, Heidelberg: Springer Berlin Heidelberg; 2012 [cited 2017 Dec 21]. p. 401–6. Available from: http://link.springer.com/10.1007/978-3-642-29210-1_64

10. Miller BG. Life table methods for quantitative impact assessments in chronic mortality. J Epidemiol Community Health. 2003;57:200–6.

11. R Core Team. R: A language and environment for statistical computing [Internet]. Vienna: R Foundation for Statistical Computing; 2019 [cited 2018 Feb 1]. Available from: http://www.R-project.org/.

12. Global Burden of Disease Study 2016 (GBD 2016) Causes of Death and Nonfatal Causes Mapped to ICD Codes | GHDx [Internet]. [cited 2020 Jun 15]. Available from: http://ghdx.healthdata.org/record/ihme-data/gbd-2016-cause-icd-code-mappings

13. Nomis - Official Labour Market Statistics - Nomis - Official Labour Market Statistics [Internet]. [cited 2020 Apr 18]. Available from: https://www.nomisweb.co.uk/query/construct/summary.asp?mode=construct&version=0&dataset=2002

14. GBD Results Tool | GHDx [Internet]. [cited 2020 Apr 18]. Available from: http://ghdx.healthdata.org/gbd-results-tool

15. Afshin A, Sur PJ, Fay KA, Cornaby L, Ferrara G, Salama JS, Mullany EC, Abate KH, Abbafati C, Abebe Z, et al. Health effects of dietary risks in 195 countries, 1990–2017: a systematic analysis for the Global Burden of Disease Study 2017. The Lancet. 2019;
